# Successful Reconstruction of Iatrogenic Pulmonary Vein Stenosis

**DOI:** 10.1016/j.jaccas.2025.106714

**Published:** 2026-01-07

**Authors:** Patrizio Lancellotti, Raluca Dulgheru, Vincenzo Miraglia, Nils De Marneffe, Cédric Davidsen

**Affiliations:** Department of Cardiology, University of Liège Hospital, GIGA Institutes, Cardiovascular Sciences and Metabolism, CHU Sart Tilman, Liège, Belgium

**Keywords:** atrial fibrillation, echocardiography, percutaneous angioplasty, pulmonary vein stenosis

## Abstract

**Image(s):**

Multimodality imaging of iatrogenic pulmonary vein stenosis (echocardiography, computed tomography angiography, fluoroscopy).

**Case Summary:**

A middle-aged woman developed progressive dyspnea after high-power short-duration ablation for atrial fibrillation. Imaging identified severe left inferior and moderate left superior pulmonary vein stenosis. Staged percutaneous stent implantation successfully restored patency in both veins, with sustained symptomatic and hemodynamic improvement on follow-up.

**Take-Home Messages:**

Pulmonary vein stenosis should be suspected in patients with unexplained dyspnea after ablation. Multimodality imaging and staged percutaneous stenting provide effective and durable management.

**Level of Difficulty:**

Advanced.

## Graphical Abstract

**Figure:**
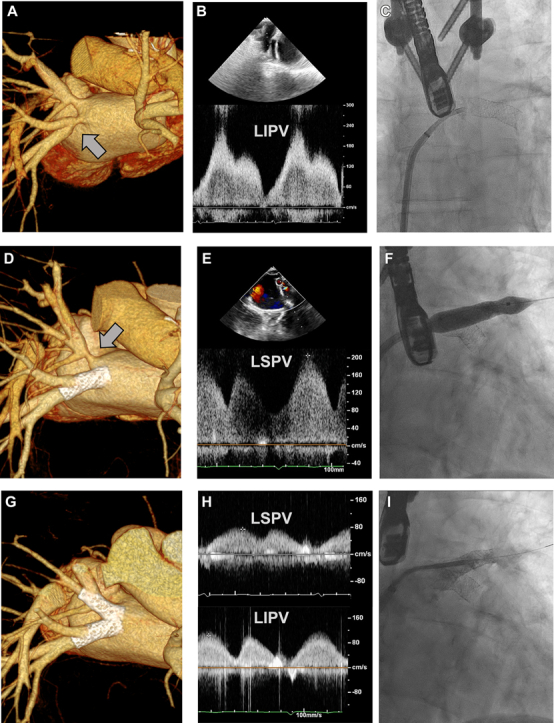

Iatrogenic pulmonary vein stenosis is a rare but potentially serious complication following left atrial or pulmonary venous interventions, often related to fibrotic remodeling and neointimal hyperplasia at the venous ostium. It has been classically associated with pulmonary vein isolation performed using thermal energy sources such as radiofrequency ablation or cryothermal energy. Although its reported incidence initially declined to below 1%,^1^ recent technological advances have renewed concerns regarding its occurrence.

The widespread adoption of contact force–sensing catheters, irrigated ablation systems, and wide antral isolation strategies may increase unintended perivenous thermal injury. In parallel, emerging evidence indicates a higher incidence of pulmonary vein stenosis with high-power and very-high-power short-duration ablation protocols,^2^^,^^3^ potentially driven by higher achieved tissue temperatures, greater thermal gradients, and deeper lesion formation extending into the venous ostium.

A middle-aged woman was referred for progressive dyspnea and reduced exercise tolerance following catheter-based ablation for atrial fibrillation using a high-power short-duration radiofrequency strategy. Physical examination and resting oxygen saturation were normal. There was no chest pain, cough, fever, hemoptysis, or syncope.

Multimodality imaging revealed a severe stenosis of the left inferior pulmonary vein and a moderate narrowing of the left superior pulmonary vein (Figure 1). Doppler echocardiography demonstrated markedly increased flow velocities in the left inferior pulmonary vein (>1.8 m/s). Computed tomography angiography confirmed tight ostial stenosis with poststenotic dilatation.

**Figure 1:**
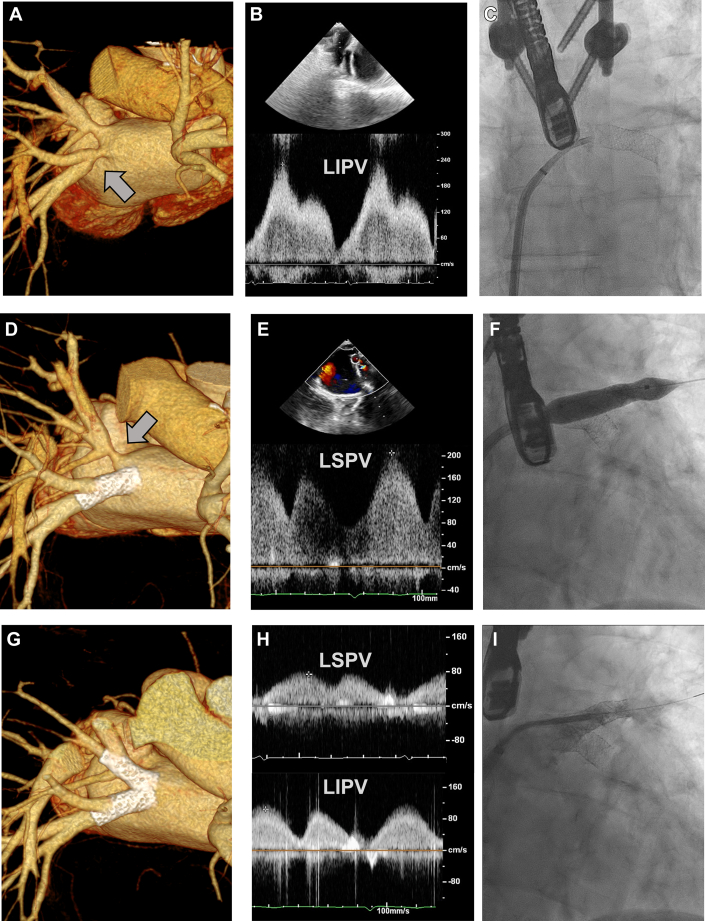
Staged Percutaneous Reconstruction of Iatrogenic Pulmonary Vein Stenosis (A) Computed tomography angiography showing severe left inferior pulmonary vein stenosis (LIPV, arrow). (B) Doppler echocardiography demonstrating elevated flow velocity in the left inferior pulmonary vein. (C) Fluoroscopic view after stent implantation in the inferior vein. (D) Computed tomography angiography showing progression of left superior pulmonary vein stenosis (LSPV, arrow). (E) Doppler velocities before superior vein stenting. (F) Deployment of superior vein stent. (G and H) Follow-up computed tomography and Doppler confirming patency of both stents. (I) Final fluoroscopic image showing 2 stents in the left pulmonary veins.

Given the hemodynamic significance of the inferior vein obstruction, percutaneous intervention was performed under fluoroscopic guidance using balloon predilatation with a Passeo 6/40-mm balloon, followed by implantation of a Dynetic 10/28-mm stent, achieving immediate restoration of luminal patency, normalization of venous Doppler velocities, and complete resolution of symptoms. Postprocedurally, the patient was discharged on dual antiplatelet therapy for 6 months.

During follow-up, the previously treated inferior vein remained patent; however, progressive obstruction of the superior pulmonary vein developed and became hemodynamically significant. A second-staged percutaneous intervention was performed, with successful stent implantation in the superior pulmonary vein using the same interventional strategy.

Postprocedural computed tomography and Doppler imaging confirmed excellent patency of both pulmonary veins with normalization of flow and pressure gradients. At 18-month follow-up, the patient remained asymptomatic with preserved left atrial function.

This vignette illustrates that pulmonary vein stenosis may progress sequentially, involving different veins over time, and highlights the importance of long-term surveillance following ablation. High-resolution imaging is essential for early diagnosis, procedural planning, and follow-up. When flow-limiting disease is present, staged percutaneous stenting can provide durable anatomical and clinical recovery, representing an effective alternative to surgical reconstruction.

## Funding Support and Author Disclosures

The authors have reported that they have no relationships relevant to the contents of this paper to disclose.

**Take-Home Messages**

• Pulmonary vein stenosis should be considered in any patient with unexplained dyspnea after atrial fibrillation ablation.
• Image-guided staged percutaneous stenting provides effective and durable treatment in multivein disease.
